# Antistreptococcic Serum in Phthisis with Cavitation

**Published:** 1908-01-18

**Authors:** 


### Points in Treatment.

### ANTISTREPTOCOCCIC SERUM IN PHTHISIS WITH CAVITATION.

It by no means follows that because a given micro-organism is found in a given place in a patient it is the cause of the symptoms from which that patient is suffering. When many bacteria are present in the same place, therefore, it is extremely difficult to determine which of them, if any, is causing detriment to the patient's health.

The above applies as strongly to the sputum as to anything else. A person may be in absolutely good health and yet his sputum may contain pneumococci, for example. Nevertheless, there is a general consensus of opinion that many of the worst symptoms of phthisis are due less to the tubercle bacilli themselves than to the other organisms, chiefly pyogenic or putrefactive, which so readily invade a lung which has been damaged by tubercle. This *secondary infection* of phthisical cavities is regarded, and rightly so, we think, as a very serious addition to the phthisis itself. The question therefore arises whether, in treating a patient who has this secondary infection, attention should not be directed quite as much to the accessory bacteria as to the tubercle bacilli.

In many institutions, and indeed in the practice of many medical men, the sputum of phthisical patients is always examined not only for the presence of tubercle bacilli, and for that very important point the presence or absence of elastic fibres which alone affords a measure of the active lung disintegration, but also for the degree to which other micro-organisms are present too. The varieties of these super-added bacteria are noted, and their relative proportions. This practice should be universal. It seems very likely that many of the pyrexial phenomena of phthisis are due to toxins absorbed from cavities in which various microbes are multiplying. One sees similar phenomena in the acute exacerbations which occur in cases of bronchiectasis, when no tubercle bacilli are present at all. If the walls of bronchiectatic cavities were as thin as those of advancing phthisis the symptoms would be very similar in the two. Fortunately, bronchiectatic cavities are usually so thick-walled that little absorption takes place through them, and for long periods at a stretch the patients may have copious expectoration and yet no fever. One sees precisely the same thing in very chronic phthisis, where the walls of the vomicæ have become dense with mature fibrous tissue; but in ordinary phthisis the granulation tissue which forms upon the walls of the cavities is broken down almost as fast as it is produced, so that it has little chance to become converted into fibrous tissue. The breaking down of the walls is partly due to the caseation of tubercles in it, of course; but it is also partly due to an ulcerative process induced by the other bacteria, precisely similar to ulceration on an external surface. It has been suggested, with some evidence to support the suggestion, that if tubercle bacilli were the only organisms growing in the lung profuse hæmoptysis from erosion of an arteriole would be rare; for a purely tuberculous process advances with comparative slowness, at any rate sufficiently slowly to allow a vessel whose wall is infected to become closed by blood clot, so that when what was the lumen is laid open, little or no hæmorrhage occurs. Ulceration by pyogenic organisms goes much faster, so that a vessel may not have time to become thrombosed before the eroding process has got into its lumen. In other words, the profuse and sometimes fatal hæmoptysis of later phthisis may be compared directly to the secondary hæmorrhage that used to be so common after operations, it being due to staphylococci, streptococci, and so forth, rather than to the intrinsically tuberculous process. Be this as it may, those symptoms of phthisis which may be summarised as "hectic" are thought by many to be due to something else than the tubercle bacillus. The question is: Which of all the organisms simultaneously present in the sputum is the worst offender?

The answer to this may perhaps be gathered from the following figures for the results of cultivations from the lung cavities in 100 cases of fatal phthisis:

| | | |
|---|---|---|
| Streptococcus ... ... ... | was present in | 74 |
| Bacillus Coli Communis ... | ,, | 74 |
| Staphylococcus Pyogenes albus | ,, | 50 |
| Staphylococcus Pyogenes aureus | ,, | 48 |
| Yeast Fungus ... ... ... | ,, | 24 |
| Sarcina ... ... ... ... | ,, | 20 |
| Bacillus Pseudo-Diphtheriæ ... | ,, | 18 |
| Pneumococcus ... ... ... | ,, | 16 |
| Bacillus, unidentified ... ... | ,, | 12 |
| Bacillus Lactis Aërogenes ... | ,, | 6 |
| Bacillus Pyocyaneus ... ... | ,, | 4 |
| Diplococcus, unidentified ... | ,, | 4 |
| Spirillum ... ... ... | ,, | 2 |

The above are all post-mortem findings, so that the bacillus coli communis can be neglected. It is present in most organs in the body some hours after death. Streptococci and staphylococci, on the other hand, are not accidental, and their occurrence in such a large proportion of the cases suggests, even if it does not prove, that they are the main offenders in connection with hectic symptoms. We have no efficient serum for the cure of staphylococcal lesions. It is true a vaccine may be prepared and used against staphylococci, and it might well be that it would be of great service in some cases of phthisis. We must, however, restrict ourselves to one point here, and we have chosen that of antistreptococcic serum.

This particular serum has been a good deal maligned, largely because it has been expected to do good in cases where streptococci have not been proved to be offending organisms. Infective endocarditis patients are cases in point. There are, however, a great many recorded instances of extreme illness due to streptococci, where the giving of serum was followed by recovery, even when all hope seemed gone. It is very difficult, of course, to prove absolutely that the serum was the cause of the recovery, but there is little doubt of the fact in the minds of those who have seen such cases.

It is not a new suggestion that antistreptococcic serum should be tried in these bad cases of phthisis with secondary infection, but this line of treatment has hitherto been comparatively little resorted to.

Those who have tried it, in sanatoria and elsewhere, are convinced that great benefit results from it in certain cases. It will not do good in every instance because it is not always the streptococcus which is at work, but if streptococci abound in the sputum antistreptococcic serum is a useful adjunct in treatment.

It acts best when given hypodermically, but it will also act when given per rectum with a tube long enough to carry it high up. This was pointed out, we believe, by Sir Dyce Duckworth. It may be administered to the extent of one phial every day or every other day in this way over a long period. Given per rectum no serum-phenomena follow. The main objection to the treatment is its cost. No ill effects accrue, and now and then very great good results, so that we would urge more extensive analyses of phthisical sputa for secondary organisms, and, if streptococci abound, treatment of the patient not only on open-air lines but also by antistreptococcic serum given per rectum.

